# Supplementary material for: Genome-Wide Analysis and Expression Profiles of Auxin Response Factors in Ginger (Zingiber officinale Roscoe)
Source: Int J Mol Sci. 2025 Aug 29;26(17):8412. doi: 10.3390/ijms26178412 (PMC12428267; doi:10.3390/ijms26178412)
Supplement: Supplementary file 1 [file ijms-26-08412-s001.zip › ijms-3756269-supplementary/Supplementary Materials/Figure S1 Sequence logos for the conserved motifs of ARF ptoreins in ginger.pdf]

ARF

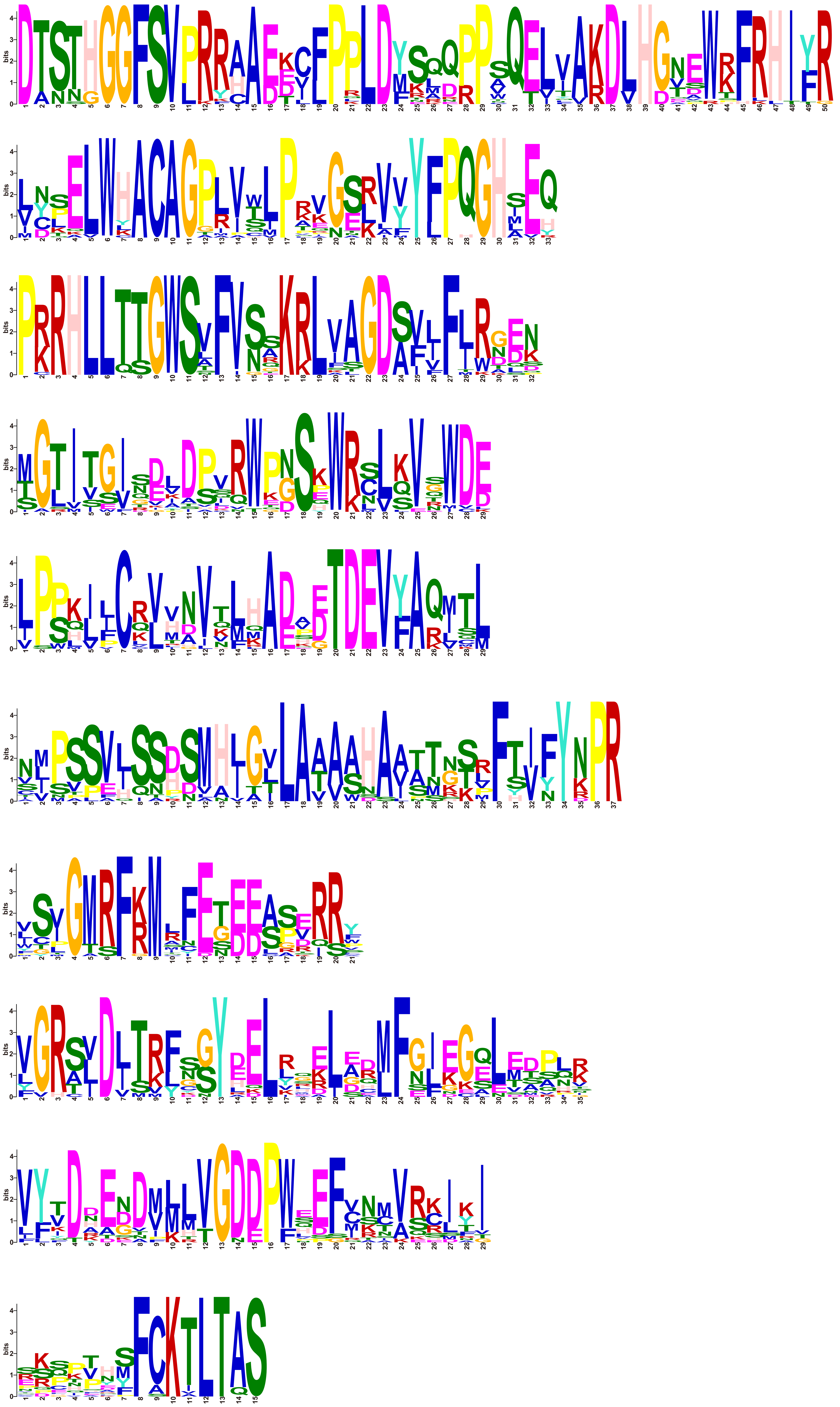

	E-value	Site	Width
motif 1	4.0e-925	25	50
motif 2	1.2e-544	25	33
motif 3	5.8e-456	22	32
motif 4	3.2e-370	24	29
motif 5	5.1e-363	23	29
motif 6	3.0e-342	19	37
motif 7	3.9e-259	25	21
motif 8	1.2e-229	18	35
motif 9	9.0e-234	18	29
motif 10	5.4e-164	26	15

Figure. S1. Sequence logos for the conserved motifs of ARF ptoreins in ginger
