# Supplementary figures and images for: Genome-Wide Analysis and Expression Profiles of Auxin Response Factors in Ginger (Zingiber officinale Roscoe)
Source: Int J Mol Sci. 2025 Aug 29;26(17):8412. doi: 10.3390/ijms26178412 (PMC12428267; doi:10.3390/ijms26178412)

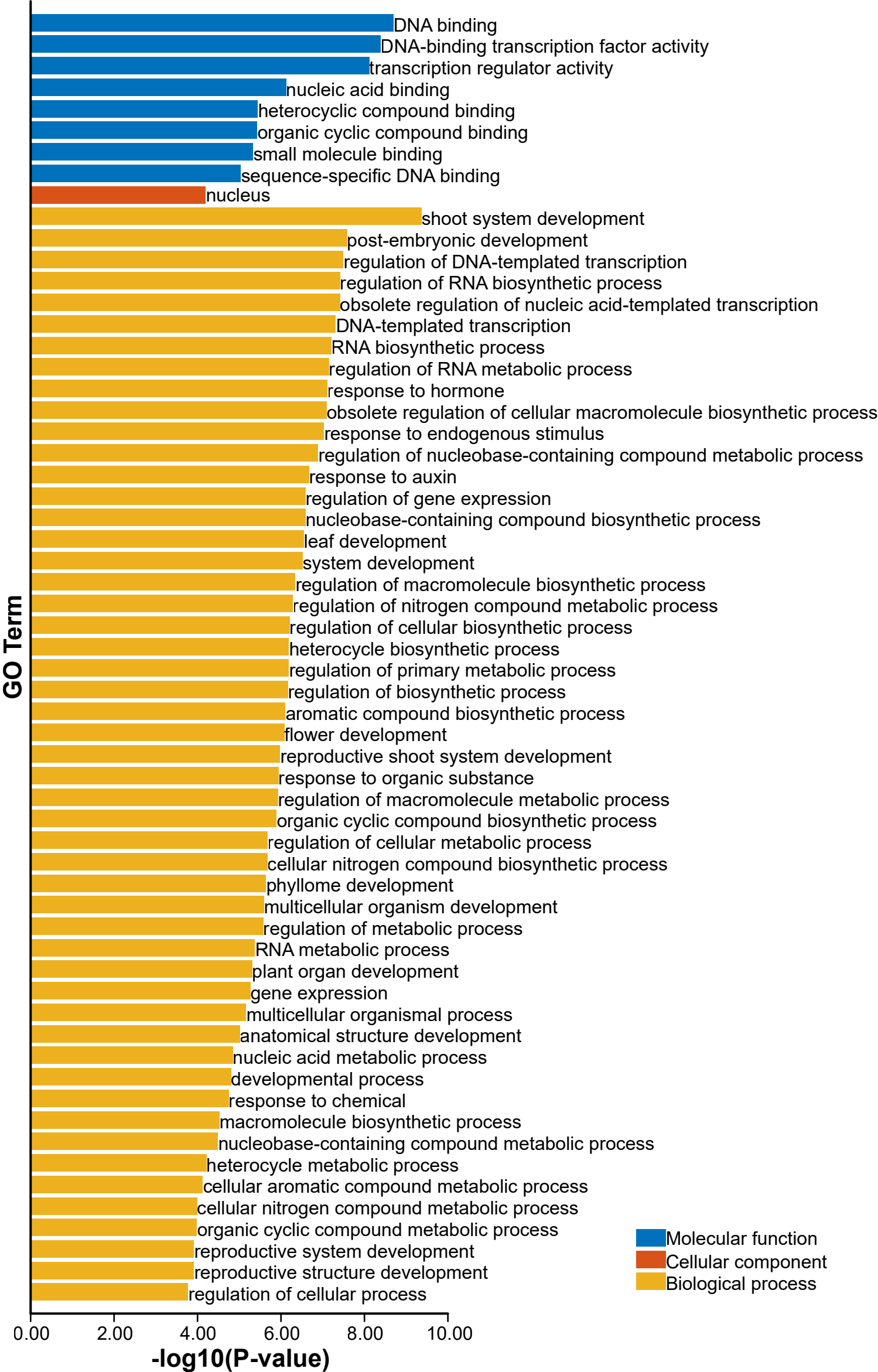

Figure. S2. GO annotation of ZoARF protein sequences

Supplement: Supplementary file 1 [file ijms-26-08412-s001.zip › ijms-3756269-supplementary/Supplementary Materials/Figure S2 GO annotation of ZoARF protein sequences.pdf]
